# Cancer Connectors: Connexins, Gap Junctions, and Communication

**DOI:** 10.3389/fonc.2018.00646

**Published:** 2018-12-21

**Authors:** Maksim Sinyuk, Erin E. Mulkearns-Hubert, Ofer Reizes, Justin Lathia

**Affiliations:** ^1^Department of Biological, Geological, and Environmental Sciences, Cleveland State University, Cleveland, OH, United States; ^2^Department of Cellular and Molecular Medicine, Lerner Research Institute, Cleveland Clinic, Cleveland, OH, United States; ^3^Cleveland Clinic Lerner College of Medicine, Cleveland Clinic, Cleveland, OH, United States; ^4^Case Comprehensive Cancer Center, Case Western University, Cleveland, OH, United States; ^5^Rose Ella Burkhardt Brain Tumor and Neuro-Oncology Center, Lerner Research Institute, Cleveland Clinic, Cleveland, OH, United States

**Keywords:** connexin, gap junction, intercellular communication, hemichannels, bystander effect, cancer, cancer stem cells

## Abstract

Despite concerted clinical and research efforts, cancer is a leading cause of death worldwide. Surgery, radiation, and chemotherapy have remained the most common standard-of-care strategies against cancer for decades. However, the side effects of these therapies demonstrate the need to investigate adjuvant novel treatment modalities that minimize the harm caused to healthy cells and tissues. Normal and cancerous cells require communication amongst themselves and with their surroundings to proliferate and drive tumor growth. It is vital to understand how intercellular and external communication impacts tumor cell malignancy. To survive and grow, tumor cells, and their normal counterparts utilize cell junction molecules including gap junctions (GJs), tight junctions, and adherens junctions to provide contact points between neighboring cells and the extracellular matrix. GJs are specialized structures composed of a family of connexin proteins that allow the free diffusion of small molecules and ions directly from the cytoplasm of adjacent cells, without encountering the extracellular milieu, which enables rapid, and coordinated cellular responses to internal and external stimuli. Importantly, connexins perform three main cellular functions. They enable direct gap junction intercellular communication (GJIC) between cells, form hemichannels to allow cell communication with the extracellular environment, and serve as a site for protein-protein interactions to regulate signaling pathways. Connexins themselves have been found to promote tumor cell growth and invasiveness, contributing to the overall tumorigenicity and have emerged as attractive anti-tumor targets due to their functional diversity. However, connexins can also serve as tumor suppressors, and therefore, a complete understanding of the roles of the connexins and GJs in physiological and pathophysiological conditions is needed before connexin targeting strategies are applied. Here, we discuss how the three aspects of connexin function, namely GJIC, hemichannel formation, and connexin-protein interactions, function in normal cells, and contribute to tumor cell growth, proliferation, and death. Finally, we discuss the current state of anti-connexin therapies and speculate which role may be most amenable for the development of targeting strategies.

## Introduction

To ensure the proper coordination of tissue function, rapid intercellular communication is required between individual cells, as well as between cells and their microenvironment (1). Under normal physiological conditions, cells respond to a number of external stimuli including soluble mediators (2), the surrounding extracellular matrix (ECM) (3), and their neighboring stroma (4). As such, cell-cell and cell-ECM interactions are integral communication mechanisms by which homeostasis is maintained, to allow for precise signaling in response to both external and internal stimuli. On a cellular level, adhesion molecules play a critical phenotypic role, as evidenced by their multifunctionality in providing structural support and mediating cytoskeletal organization (5). Furthermore, adhesion complexes, including adherens junctions, tight junctions, and gap junctions (GJs), are necessary for the initiation and integration of signaling cascades that may seem unrelated to their canonical function (6).

Disruption of adhesion complexes has typically been understood to interfere with normal tissue function and serves as the initiating event for pathophysiological disorders. Among such mechanisms, intercellular communication mediated by GJs has been found to be vital for the maintenance of cell survival in a variety of different tissues (7). Electron microscopy analyses revealed that GJs often present as distinct crystalline-like plaques on cell membranes that are composed of a family of proteins termed connexins. Thus, far, 20 different connexin genes have been characterized in mice and 21 in humans (8). Each connexin has tissue- and developmental specific functions in mammalian biology, although redundancy does exist between subunits (6). This has been experimentally demonstrated, as different connexin isoforms display spatial and temporal specificity, which is modulated by transcription factors including the Sp transcription factors (Sp1 and Sp3), activator protein (AP-1), and members of the Jak/STAT pathway (9). Furthermore, cell-specific transcription factors such as Nkx2, HNF-1, Mist1, and NF-κB, among others, can regulate connexin gene expression, allowing for precise expression of connexins during development and homeostasis (10).

To distinguish individual subunits, connexin proteins are designated by a molecular mass, while their respective genes are classified by a sequence homology at the nucleotide and amino acid levels. Accordingly, at least three subgroups of connexins have been described and are classified as α, β, or γ. Thus, a 26 kDa connexin protein is referred to as connexin 26 (Cx26) or gap junction β-2 (GJB2). Structurally, all connexin protein subunits have been shown to share a comparable topology, composed of cytoplasmic N-, and C- terminal domains along with four transmembrane regions, two extracellular loops, and one intracellular loop (11). However, different isoforms exhibit variability in their cytoplasmic domains, which allow for a variety of different interactions and biological roles (12). Most connexins are modified posttranslationally through phosphorylation, primarily on serines, which regulate a variety of connexin processes such as trafficking to membranes, assembly, degradation, and gating of functional GJ channels (13). During their short half-life of ~2–4 h, six connexin proteins form a hexameric arrangement in the endoplasmic reticulum or Golgi body and are then trafficked as connexons, or hemichannels, to cellular membranes (14). Connexons can be composed of the same connexin subunit to form homomeric connexons or different subunits to form heteromeric hemichannels. However, not all connexin combinations are capable of forming functional channels, and not all channels have an equal capability to dock with one another (15). Thus, specific arrangements confer different properties of conductance and regulation in the resulting channels, which allows for a level of control for intercellular communication.

To mediate cell-cell communication, connexons from one cell dock with connexons of adjacent cells, forming GJ intercellular channels that allow the passage of ions, second messengers, microRNAs (miRNAs) (16), and other small molecules directly between the cytoplasm of joined cells, without contacting the extracellular environment (17). This allows cells to quickly coordinate their behavior and regulate signaling during development and normal physiology in various organs including the brain, heart, eyes, liver, ovaries, breasts, and skin, among others (18). The function of connexins and, by extension, gap junctional intercellular communication (GJIC) is of critical importance for normal physiology as evidenced by the ubiquitous expression of connexin proteins in nearly every mammalian cell [summarized in Goodenough et al. (19)]. Furthermore, many cell types co-express two or more connexins that may have overlapping or distinct functions. For example, keratinocytes have been shown to express Cx26 (20), Cx43 (21), Cx31.1(22), and Cx30 (23). Additionally, cardiomyocytes have been found to express Cx40 (24), Cx43 (25), and Cx45, while hepatocytes primarily express Cx26 and Cx32 (26). In this manner, co-expression of multiple connexin family members within the same cell type allows for compensatory communication mechanisms, should the expression of one subunit become perturbed.

Historically, studies of connexin function have focused on their role in the formation of GJs to enable GJIC between cells. However, during the 1990s, evidence began to emerge suggesting an alternative role for GJs, in the form of undocked hemichannels [covered in Goodenough and Paul (27)]. It was thought that undocked connexin hemichannel activity would drown cells in Na^+^ and Ca^2+^ and lead to the loss of metabolites necessary for cellular function. Open hemichannels have been described in Xenopus oocytes, mediated in part by Cx46 (28). Conversely, it was also found that oocytes rapidly deteriorated and died unless high amounts of Ca^2+^ were present to maintain the hemichannel in a closed state. Thus, hemichannel opening and closing was determined to be a dynamic process that enables the ingress or egress of cytoplasmic contents and extracellular material. Further studies found that Cx44 (29) and Cx56 (30) are also able to form conductive hemichannels in Xenopus oocytes, while other subunits such as Cx35 (31), Cx32 (32), and Cx52.6 (33) were also later characterized to have similar capabilities. Thus, a second important role of connexins has quickly become apparent and warrants closer scrutiny, which we will provide in this review.

Lastly, regulation of GJIC can be modulated by connexin-associated proteins including regulatory phosphatases, cytoskeletal elements, and enzymes. Interacting partners include zona occludens 1 (ZO-1) (34), v-Src (35), pkaC (36), cadherin (37), caveolin (38), and MAPK (39), among many others. Additional connexin-interacting partners are also likely to exist but have not been characterized due to the number of connexins and the diversity of their C-terminal domains. Thus, apart from facilitating GJIC and hemichannel activity, GJs have increasingly been perceived as signaling complexes that are important for the regulation of cell function and transformation (1). As such, a complete understanding of connexin biology and subsequent GJ function can only be achieved through the identification of the binding partners that may play critical roles in GJ formation, gating, and transport (40, 41).

**Figure 1:**
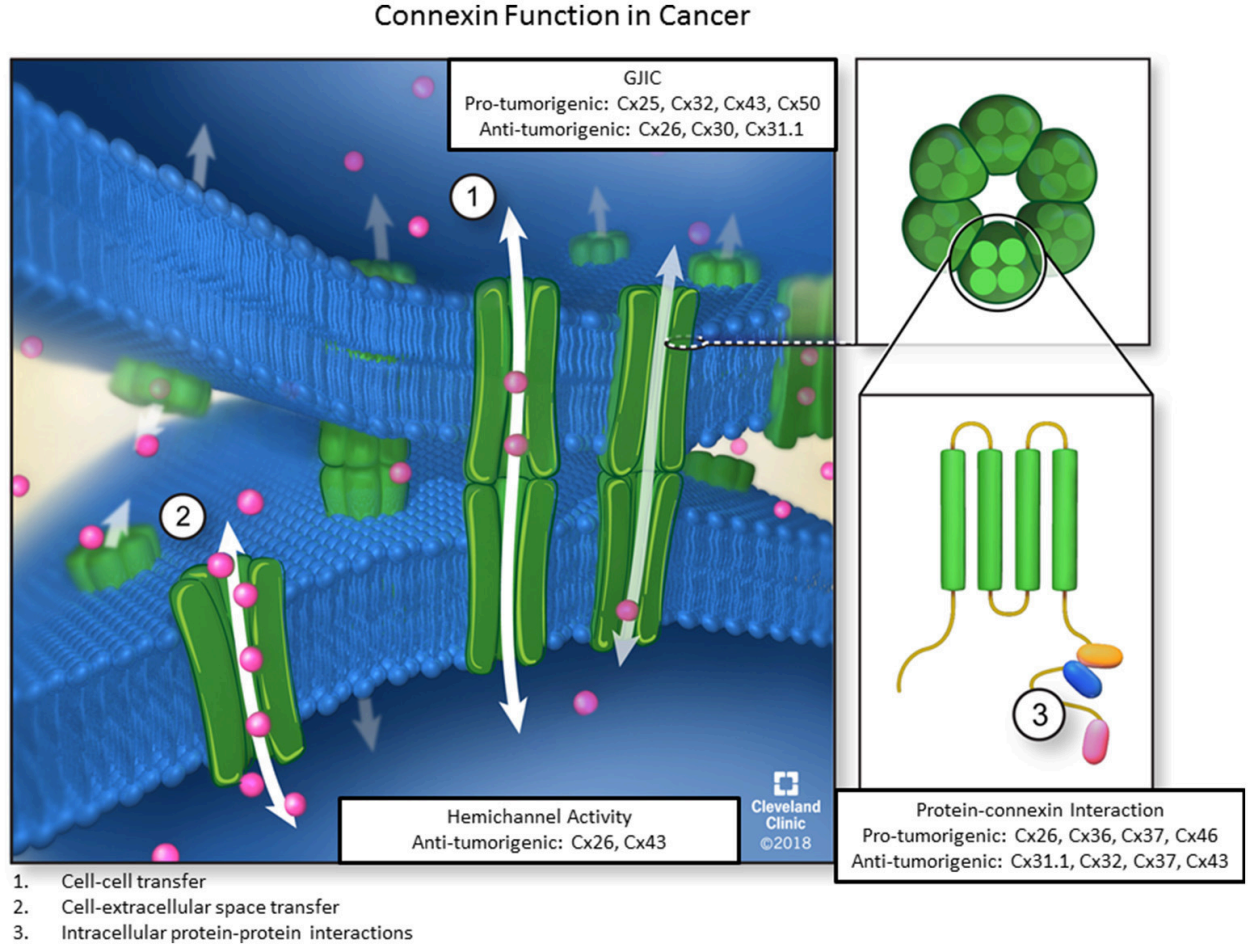
The three main functions of connexins. Six connexin subunits are able to oligomerize into membrane-spanning structures termed connexons. Connexons from adjacent cells are capable of docking and forming channels through which ions, second messengers, miRNAs, and other small signaling molecules can passively diffuse between coupled cells without contacting the extracellular environment. Furthermore, individual connexons can function as hemichannels to allow molecules from the ECM to enter or exit the cellular cytoplasm via diffusion. Lastly, connexin subunits have an intracellular C-terminal domain, allowing for connexin-protein interactions and impacting downstream signaling events via GJ-independent mechanisms. Each of the three functions also includes pro-or anti-tumorigenic roles for different connexin subunits.

Consequently, connexin biology can be broadly classified by three different criteria, namely cell-cell communication, hemichannel activity, and direct connexin-protein interaction, to activate signaling pathways and affect cellular phenotypes (Figure 1). Each function plays a distinct role in normal physiology and is necessary for proper cellular behavior during development, as connexin dysfunction in each of the described axes can contribute to a wide variety of disease states including cancer. Thus, it is critical to understand connexin multifunctionality in normal physiology and pathological states. The purpose of this review is to characterize connexins in the context of each of the three canonical roles and describe how dysfunction of each distinct connexin role, can affect cellular phenotypes in pathophysiological conditions, particularly cancer.

## Connexin Mutations and Disease

Multicellular organisms require intercellular communication to coordinate complex behavioral mechanisms and utilize GJ channels as a common means of intercellular communication. It is therefore not surprising that connexin dysfunction is associated with disease states. In addition, to better understand and appreciate the role of connexins and GJIC in the context of cancer, a brief review of connexins in disease states, unrelated to neoplasms, is necessary to demonstrate how aberrant GJs, dysfunctional hemichannels, or lack of proper protein signaling contributes to pathogenesis.

There are currently over two thousand peer-reviewed articles implicating connexins in a wide variety of pathologies, and as such, it is virtually impossible to cover each example in a single review. Due to their functional specificity, cells are required to tightly control connexin expression during all stages of development and homeostasis. When this process goes awry, as a result of heritable or acquired mutations, aberrant connexin expression can be associated with a variety of pathologies. However, mutations in connexin genes that result in disease have diverse effects on connexin protein expression. In some cases, mutant connexins do not move past quality control mechanisms and are thus forced into endoplasmic reticulum-associated degradation (ERAD) or become arrested in the Golgi apparatus [summarized in Laird et al. (42)]. Mutated connexins can also lose the ability to complex into functional hemichannels or GJs due to dysfunction in the channel pore. A brief summary of connexin mutations and their consequences in a variety of diseases is provided in Table 1. Likewise, in some cases, connexin mutations can result in proteins acquiring an aberrant half-life, increasing the turnover before it can complete its normal function. Some mutations can also cause connexins to lose their ability to associate with the interactome, leading to disease formation. Lastly, mutations can result in gain-of-function mechanisms that cause affected connexins to oligomerize with subunits that they would not normally interact with. The resulting aberrant interactions can lead to improperly activated hemichannels as well as dead or leaky GJ channels, which can contribute to cellular pathologies (42). The disruption of each of the three main functions of connexins can therefore drive the development of pathological conditions.

**Table 1:** Genetic disorders resulting from connexin mutations.

**Connexin**	**Gene**	**Disease**	**References**
Cx26	*GJB2*	Deafness, keratitis-icthyosis-deafness syndrome	(43, 44)
Cx30	*GJB6*	Non-syndromic hearing loss	(45)
Cx30.3	*GJB4*	Erythrokeratoderma variabilis	(46)
Cx31	*GJB3*	Erythrokeratoderma variabilis	(47)
Cx31.3	*GJC3*	Non-syndromic hearing loss	(48)
Cx32	*GJB1*	Charcot-Marie-Tooth neuropathy, X-linked 1	(49)
Cx40	*GJA5*	Atrial fibrillation	(50)
Cx43	*GJA1*	Oculodentodigital dysplasia, keratoderma-hypotrichosis-leukonychia totalis syndrome.	(51, 52)
Cx45	*GJC1/GJA7*	Atrial arrhythmia	(53)
Cx46	*GJA3*	Congenital cataracts	(54)
Cx47	*GJC2/GJA12*	Pelizaeus-Merzbacher-like-disease type 1	(55)
Cx50	*GJA8*	Autosomal dominant congenital cataract	(56)

*Connexin mutations result in a variety of different diseases, most notably deafness and skin disorders. Additional abnormalities include congenital cataracts, atrial fibrillation, and neurological dysfunction in the form of demyelinating disease*.

### Connexins of the Heart

Cardiac cells are known to contain several connexins in addition to Cx43, namely Cx40, and Cx45 (57, 58). Moreover, disruption of GJIC and hemichannel activity has been thought to play a role in a variety of different cardiac pathologies, resulting in both electrical disturbances and structural abnormalities. Each of the three connexin genes have been deleted via embryonic stem cell targeting, and it has been shown that all three genes are necessary for heart conduction (59). As such, conditional deletion of Cx43 in adult myocardiocytes only, impacted heart conduction and suggested that a lack of Cx43 could induce an arrhythmogenic phenotype, which can contribute to heart dysfunction (60, 61). Complete knockout of Cx45 or Cx43 in mice leads to early death during gestation, due to a conduction block, endocardial cushion defects, or cardiac malformation (62, 63). In contrast, Cx40 knockout mice are embryonically viable but show evidence of slowed conduction and a partial atrioventricular block (64). Furthermore, knock-in gene replacement studies, during which the coding region of the *GJA1* gene encoding Cx43 was replaced by the coding regions of Cx32 or Cx40, rescued the embryonic lethality of Cx43-deficient mice (65). Importantly, it was also found that animals with Cx43 replacement exhibited mild tissue morphological abnormalities, demonstrating that each connexin subunit has a different function depending on its resident cell and tissue (65). Originally, uncoupling of GJs and the inhibition of GJIC was thought to have a beneficial effect on cardiac cells, by preventing the spread of tissue damage. However, in direct contrast to this assumption, later studies found that uncoupling cardiac cells with a broad-spectrum GJ inhibitor, heptanol, resulted in a decrease in arrhythmia scores during ischemia and reperfusion. In addition, infarct size due to ischemia was reduced, and heptanol-mediated uncoupling was thus shown to confer cardioprotective effects in a rat model of cardiac cell death (66). Connexin-protein interactions have also been implicated in cardioprotection to regulate cardiomyocyte mitochondrial function and metabolism. Through immunoprecipitation and mass spectrometry, Cx43 was described to interact with an apoptosis-inducing factor (AIF) and the β-subunit of the electron-transfer protein (ETFB) to regulate mitochondrial respiration and reactive oxygen species (ROS) generation (67). Thus, all three functions have been described in heart tissue, indicating that multiple communication mechanisms, mediated by connexins, exist for the regulation, and development of cardiac cells.

### Connexin Expression and Neurological Disorders

GJIC and hemichannel activity have also been found to contribute to diseases of the nervous system. Within the mammalian peripheral nervous system, GJs are mainly associated with myelinating Schwann cells. Cx32 forms GJs between the myelin lamellae, connecting the Schwann cell cytoplasm with the adaxonal cell compartment inside the myelin sheath (68). This arrangement allows for the diffusion of ions and small molecules across adjacent cell membranes, which form the myelin sheath. Thus, Cx32 plays a crucial role in the maintenance and homeostasis of myelinated axons by forming functional GJs (57). Indeed, mutations in Cx32 were implicated in human disease, namely Charcot-Marie-Tooth neuropathy X type 1 (CMTX1), a progressive peripheral neuropathy defined by a mixture of demyelination and axonal degeneration (69). More than 400 mutations have been found in the *GJB1* gene encoding Cx32, while both *in vitro* and *in vivo* models of the disease confirm that most Cx32 mutations result in the inability of the connexin to form a functional GJ (70). Likewise, mutations in Cx32 were found to induce an abnormal hemichannel opening, ostensibly causing excessive plasma membrane permeability and subsequently affecting cell survival (71). Connexin hemichannels have increasingly been implicated as key players in spreading ischemic brain injury through the propagation of cell death messages in the form of ATP, NAD^+^, or glutamate as a result of abnormally prolonged openings, and subsequent loss of intercellular contents [reviewed in Davidson et al. (72)] In addition, oligodendrocytes, the main myelin sheath-creating cells in the CNS, have been found to express Cx32, Cx29/31.1, and Cx47. Loss of both Cx32 and Cx47 was further associated with severe CNS demyelination and mortality in mice (73). As such, GJ and connexin hemichannel function are well described in CNS disorders, although the exact molecular mechanisms remain under investigation [reviewed in Xie et al. (74)]. Thus, the identification of functional connexin activity in the CNS provides further interest for their role in neurological disorders and makes essential information available for the development of therapeutic approaches that arise as a result of dysfunctional intercellular communication.

### Connexins, Communication, and Deafness

While connexin gain-of-function mutations feature prominently in skin diseases, the opposite is true of autosomal-recessive, nonsyndromic, sensorineural deafness. Interestingly, over 90 unique genes overall have been found to be associated with deafness, although mutations in *GJB2*, which encodes Cx26, are thought to account for almost 50% of all hearing loss cases, from severe to profound (57). Mutations in Cx30 have also been found to be associated with deafness; however, such instances are much less frequent compared to Cx26 alterations. Most Cx26 mutations correlated with deafness are deletions, truncations, and frameshifts, indicating that hearing loss is mainly a result of loss of GJIC or improper hemichannel activity (75). However, there are a variety of dominant, missense mutations that concomitantly produce functional GJs and lead to both deafness as well as skin dysfunction, such as Keratitis-ichthyosis-deafness syndrome (76). Cx26 and Cx30 are mainly expressed in the supporting mechanosensory hair cells in the organ of Corti as well as in the lateral wall, which contains the stria vascularis (77, 78). The latter is necessary for the production of endolymph and generation of the endocochlear potential (EP), which is required for proper signaling in hair cells and subsequent auditory function (79). Cx26 and Cx30 are expressed in wide overlapping patterns and demonstrate abundant GJIC, indicating that extensive networks are present in the hair cells. Interestingly, Cx26 expression has also been found in basolateral and apical areas of supporting cells, specifically in regions lacking cell-cell contacts, suggesting that cells may be able to use hemichannels as a means of communication with the perilymphatic and endolymphatic compartments (80). Furthermore, multiple studies utilizing genetic ablation and transgenic approaches against Cx26, have shown that its loss of function, even when only limited to supporting epithelial cells, can lead to the death of hair cells, resulting in hearing loss (81–83). However, it is not completely clear how mutations in Cx26 and Cx30 function in tandem to result in deafness. As such, double Cx26^+/−^/Cx30^+/−^ heterozygous animals were developed and shown to exhibit EP reduction and hearing loss, although their cochlea displayed normal physiology (43). This was found to be in direct contrast to Cx26^−/−^ mice, which displayed aberrant cochlear development. Moreover, Cx26^+/−^ or Cx30^+/−^ animals showed no hearing loss or EP reduction, suggesting that only digenic Cx26 and Cx30 mutations impair coupling in the cochlear lateral wall and lead to deafness (43). Interestingly, connexin-protein interactions have also been described, which may impact the development of deafness. The C-terminus of Cx26 has been shown to interact with the GTPase effector domain of dynamin 2 (Dyn2), for example. Genetic inhibition of Dyn2 resulted in reduced Cx26 degradation, implicating it in the regulation of Cx26 endocytosis and resulting GJIC (84). Accordingly, in deafness, disruptions in each of three basic roles of connexins have been described to result in pathology, even when normal cochlear development is not affected. Thus, it is necessary to consider each purpose independently to delineate how connexins interact with each other as well as their binding partners to facilitate hearing loss.

## Cancer and Connexins

Dysfunction in connexin biology via mutations can lead to a wide variety of pathophysiologies due to altered hemichannel activity or aberrant GJ formation. In cancer, connexins were historically thought to act as tumor suppressors, as early studies interrogating liver tumor cells found that intercellular communication was absent, suggesting that cancer cell coupling may be anti-tumorigenic (85). Follow-up studies investigating connexin and GJ levels in additional tumor cell types as well as *in vivo* tumors likewise concluded that connexins have broad tumor suppressive properties (86, 87). Studies characterized intercellular communication in the context of cancer utilized rat C6 glioma cells, which are known to express low levels of Cx43. Moreover, the ability of these cells to dye couple was found to be deficient, indicating that their communicative ability was impaired (88). However, when intracranially implanted into rat brains, C6 cells were able to give rise to large gliomas. When tumor cells were transfected with full-length cDNA encoding Cx43, it was shown that dye coupling positively correlated with Cx43 expression, and these cells exhibited decreased cell growth compared to non-transfected controls (89). In addition, Cx43-transfected C6 glioma cells were found to be less tumorigenic *in vitro*, with growth rates that were inversely related to the amount of Cx43 expressed (90), demonstrating that connexin expression may be associated with decreased brain tumor growth. Cx43 was also found to be reduced in high-grade glioblastoma (GBM) samples compared to low-grade astrocytomas or mesial temporal lobe epilepsy samples, which correlated with decreased levels of GJIC (91). As chemotherapy and radiation are among the most common treatment approaches for cancer, it is useful to consider how the connexin function is implicated in tumor cell survival after exposure to the cytotoxic agents. To that end, Cx43 was found to promote temozolomide (TMZ) resistance, as Cx43 levels were inversely correlated with GBM TMZ sensitivity as well as patient survival (92). Through their communicative capacity, tumor cells are therefore able to mitigate chemotherapeutically induced cell death and protect themselves from harm. Transgenic mice lacking Cx32 (93) or Cx43 (94) have also been reported to demonstrate an increased likelihood of tumorigenesis as a result of radiation or chemical induction, supporting the hypothesis that connexins have tumor suppressive capabilities. In addition, astrocytoma cells have been shown to extend long membrane protrusions, or microtubes, to enable brain invasion and proliferation. The resulting network was also found to protect microtube-connected astrocytoma cells from radiotherapy, while this was not seen in their unconnected counterparts, demonstrating that GJIC plays a critical role in brain tumors (95). In all instances, it becomes critical to understand whether targeting the three main roles of connexins can serve as an anti-tumor therapy and which function is most amenable to selectively target cancer cells. A summary of different connexin subunits and their pro-or anti-tumorigenic activity as it relates to GJIC, hemichannel function, or protein connexin-interaction has been provided in Table 2.

**Table 2:** Three main functional roles for connexins.

**Connexin**	**Cancer**	**Function**	**Tumor Activity**	**References**
Connexin 25	Leukemia	GJIC	Pro-tumorigenic	(96)
Connexin 26	Breast, Cervical,	GJIC	Anti-tumorigenic	(97)
	Cervical	Hemichannel activity	Anti-tumorigenic	(98)
	Breast	Protein-connexin interaction	Pro-tumorigenic	(99)
Connexin 30	Glioma, Gastric	GJIC	Anti-tumorigenic	(100, 101)
Connexin 31.1	Head and neck squamous cell carcinoma	GJIC	Anti-tumorigenic	(102)
	Non-small cell lung cancer	Protein-connexin interaction	Anti-tumorigenic	(103)
Connexin 32	Breast	GJIC	Pro-tumorigenic	(104)
	Renal cell carcinoma, Ovarian	Protein-connexin interaction	Anti-tumorigenic	(105, 106)
Connexin 36	Cervical	Protein-connexin interaction	Pro-tumorigenic	(107)
Connexin 37	Liver, Insulinoma	Protein-connexin interaction	Pro-tumorigenic and anti-tumorigenic	(108, 109)
Connexin 43	Brain	GJIC	Pro-tumorigenic	(110)
	Breast	Hemichannel activity	Anti-tumorigenic	(111)
	Ovarian	Protein-connexin interaction	Anti-tumorigenic	(112)
Connexin 46	Brain	GJIC	Pro-tumorigenic	(113, 114)
Connexin 50	Cervical	GJIC	Pro-tumorigenic	(115)

*Different connexin subunits are associated with GJIC, hemichannel activity, or protein-connexin interaction in order to exert pro-or anti-tumorigenic activity*.

### Connexin Expression and Cancer

Our understanding of GJIC and connexins in cancer has grown considerably and has proven more complex than originally hypothesized. Connexins are now accepted to have a wide range of functions in addition to their tumor suppressive roles. For example, in mouse melanoma cells, it was reported that Cx26 expression played a critical role in the intravasation and extravasation of tumor cells via heterologous GJ communication with endothelial cells, linking connexin expression with invasion (116). Additionally, in multiple prostate cancer cell lines, Cx43 expression was correlated with tumor cell migration (117). Thus, far from simply acting as a repressor of tumor growth, high connexin expression can also be associated with a poor prognosis.

While connexin expression has been found to correlate with increased or decreased cancer cell growth, depending on both the neoplasm and connexin subunit, there is still a lack of understanding of exactly which molecules are exchanged between tumor cells. Given the size of the connexin family it difficult to pinpoint their exact roles in cancer biology as over 20 connexin subunits are known to exist in humans, and each may have differential roles in tumor initiation, progression, and/or metastasis. Thus, it is imperative to consider each member of the family in the context of the specific cancer type and its particular functional role as it relates to the tumor cell phenotype. In addition, it is important to remember that epidemiological studies that compare functional connexin status to the onset of cancer and its progression, are largely absent (57). As such, while connexin expression can be linked to patient prognosis, it is challenging to study the temporal distribution of connexin-mediated signaling and GJIC through the course of cancer development and growth. It is also largely unknown how mutations in connexin proteins, that lead to pathophysiological conditions other than cancer, may inform cancer risk. For example, as mentioned above, mutations in Cx26 are one of the leading causes of sensorineural hearing loss (118). However, there are no reports that organs with high Cx26 expression and resulting potential mutations, such as the liver, gallbladder, or colon, are at an increased risk of developing cancer (119). That is not to say that such connections do not exist but demonstrates that an epidemiological gap exists between connexin expression and cancer risk. It is still being investigated whether connexins represent a practical target for the prevention or treatment of cancer. While some connexins, in particular Cx43, are being studied to aid in the repair of chronic wounds to reduce edema, inflammation, and lesion spread (120), there are no current clinical trials relating to connexin function or GJIC in cancer. Indeed, caution should be used when targeting connexins in cancer, as potentially harmful secondary effects on normal tissue function could result. However, as technology advances and personalized medicine becomes more widespread, targeting specific functions of connexins as an adjunct anti-tumor strategy could have promising therapeutic value.

### Connexins and Cancer Stem Cell Function

Multiple tumor types are composed of a heterogeneous population of cells with a small tumor-recapitulating subset at their hierarchical apex, termed cancer stem cells (CSCs) [reviewed by Dick (121), Lathia et al. (122), Batlle and Clevers (123)]. As such, it is of little surprise that CSCs also require GJIC in order to proliferate and maintain their self-renewal properties through the transfer of molecules, including non-coding RNA and exosomes (124). In addition it was shown that in hepatocellular carcinoma, Cx32-mediated GJIC was critical for the expansion and self-renewal of CSCs (125). In other tumors characterized by the presence of CSCs, such as GBM (92, 113, 114, 126), and liver cancer (127), it was found that GJIC was an integral part of CSC function and resulting tumor progression. In glioma, it has been demonstrated that connexins are capable of sustaining CSC proliferation and self-renewal in a GJIC-independent manner (128). Cx43 expression, in particular, was found to be decreased in CSCs as a result of hypermethylation in the promoter region of *GJA1*. When functional Cx43 was introduced, CSC self-renewal, invasive capability, and tumorigenicity were inhibited via E-cadherin, which regulates the activation of the Wnt/catenin signaling pathway (128). Thus, CSCs may require decreased expression of Cx43 to maintain “stemness,” while upregulation of this connexin could represent a promising new strategy for the treatment of GBM. Likewise, in triple-negative breast cancer, it was found that Cx26 expression was higher in CSCs, and promotes self-renewal by forming a signaling complex with NANOG, a pluripotency transcription factor, and focal adhesion complex. Thus, the resulting complex was responsible for the stabilization of NANOG and subsequent FAK activation, promoting CSC proliferation and tumorigenicity (99). In addition, cytoplasmic accumulation of Cx32 in hepatocellular carcinoma CSCs was found to enhance self-renewal and expansion, although the exact mechanisms remain under investigation and could be the result of either GJIC, hemichannel activity, or connexin-protein interactions in the cytoplasm (125). However, additional work is critical to determine whether particular connexin subunits and functions are responsible for aberrant CSC phenotypes that contribute to tumor growth and recapitulation.

### GJs and Cancer Therapy

Given their ubiquitous nature, it is important to understand whether connexins could serve as a beneficial target for anti-tumor therapy, either by suppressing or increasing their expression and subsequent function. While loss of GJIC is often thought of as a marker for early-stage tumors, this is not an effective prognostic indicator to reliably demonstrate efficacy (87). This is because it is not completely evident which individual connexin subunits may be targetable in a particular cancer, as few mimetic peptides are currently under consideration (129). When considered along with combinatorial therapy, there is reason to believe that connexins are promising molecular targets. Of particular note is the role of the “bystander effect” in facilitating the transfer of damaged signals between adjacent cells. Briefly, one targeted cell can spread radiation and chemotherapy to a population of coupled tumor cells, thus minimizing the damage to normal cells that are not capable of communicating with their malignant counterparts. Due to these properties, the bystander effect may be a promising mechanism by which drug delivery systems can be designed to specifically target cancer cells while not affecting healthy tissue; this will be further described in a later section. However, a necessary factor involved in connexin regulation is the development of genetic protocols and chemicals that are able to induce or inhibit specific connexins in individual neoplasms or in even more complex situations such as individual tumor cells. This is particularly relevant as systemic upregulation of connexins in normal host tissues that undergo continual renewal could have harmful effects for the development of cancer (87). Enhanced connexin expression may also lead to increased cancer cell metastasis (130–132). Therefore, it becomes necessary to define which connexins play a role in normal development and which display communication or other roles in late-stage tumor progression for a given tissue. It is possible to imagine that a strategy may be developed through the use of genetic inhibition to manipulate connexin function and combine it with other therapeutic means to provide overall benefit to cancer patients.

## GJIC and Cancer

The ability for cells to communicate and exchange ions through low-resistance pathways was first described in myocardium, as adjacent cells were found to be capable of transmitting electrical synapses amongst each other (133). In 1953, visual evidence for the existence of GJ structures was demonstrated via electron microscopy in squid and crayfish Schwann cells (134). In the subsequent decades, it has been found that GJs are unique structural components of cellular plasma membranes that facilitate communication between adjacent cells (135). Accumulating evidence has demonstrated that most cells and tissues of the body utilize GJs as a means to communicate during development and normal physiology that can be co-opted by cancer cells (136). As mentioned above, GJ channels can consist of different connexons, which are themselves made up of identical or differential connexin subunits that regulate their physiological properties, conductive capacity, and permeability (137, 138). The central function of GJIC is to share metabolic demands across multiple cells in order to control spatial gradients of nutrients and signaling molecules found in the extracellular environment or that occur as a result of cell stress, which is also vital for tumor cell survival. In addition, GJIC can help cells respond to somatic mutations when a critical metabolic enzyme or ion channel becomes dysfunctional as the loss of one such biologically vital function in a particular cell can be compensated for by its neighboring counterparts (136). A particular example of such offsetting activity is Lesch-Nyhan syndrome, which results from impaired activity of hypoxanthine phosphoribosyltransferase (HGPRTase), a necessary enzyme in the nucleotide salvage pathway encoded by the HPRT1 gene (139). Dysfunction in HGPRTase results in overproduction of uric acid, causing dystonia, gout, and self-mutilation. However, when mutant fibroblasts from patients with Lesch-Nyhan were cultured with normal cells, the rescue of GJ formation was sufficient to reverse the phenotype, in a process coined as metabolic cooperation (140). Metabolic cooperation is also thought to play an important role in heterozygous female Lesch-Nyhan carriers, as HPRT1 is located on the X chromosome causing random X-inactivation which can lead to differential populations of cells that are mutant and normal. Thus, females with HPRT1 mutations are largely asymptomatic due to metabolic rescue of mutant cells by adjacent wild-type cells (141).

Communication between cells mediated by GJs is usually associated with beneficial correlatives enabling coordinated behavior and rapid response to a wide variety of differential catalysts. In other words, the ability of coupled cells to act in a concerted manner amongst each other may serve as a mitigating factor to distribute stressors and damage responses in a given tissue, minimizing the burden on individual cells (142). GJIC is also thought to have a protective role in normal physiology. However, in some instances, intercellular communication via GJs is a double-edged sword that can be potentially harmful. While the exchange of metabolites, secondary messengers, and electrical signals may have benefits for both donor and acceptor cells in response to stimuli, accumulating evidence has also suggested a potentially detrimental “side-effect” of GJIC.

Following the discovery of X-rays by Wilhelm Röntgen, subsequent studies quickly uncovered their cytotoxic and carcinogenic nature (143). Studies also showed that ionizing radiation was generally damaging to cells due to its ability to generate single- and double-strand breaks in DNA. The utility of using radiation to destroy rapidly proliferating cancer cells was quickly recognized and heralded as a new era of cancer treatment in the form of radiotherapy. In its infancy, radiation was only thought to be lethal to those cells that were directly exposed to high-energy particles. In the past two decades, it has been demonstrated that intercellular communication can promote DNA damage responses through a phenomenon termed the “bystander effect” (144). This process describes the mechanism by which intercellular communication, usually mediated by GJs, is able to induce DNA damage in those cells that have not been directly irradiated but rather have been in contact with those that have (145). Thus, it is becoming more widely recognized that direct irradiation may not be required to force cells into a DNA damage response state, especially when considered in the context of cancer. As shown by the wide variety and seemingly opposite nature of direct cell-cell communication on cell survival, the complexity of GJIC should not be underestimated. As a general rule, the inhibition of communication is most often associated with harmful cellular phenotypes due to decreased responsiveness to external and internal stimuli. Furthermore, perturbation of GJIC during development can negatively affect normal tissue function, and in many cases, mutations in connexin subunits can lead to lethality. Under baseline conditions GJIC enables cells to rapidly respond to a huge variety of different signals in order to adjust their own internal conditions to best suit the ever-changing extracellular milieu. Similar to interactions between individual people, proper communication at the cellular level is critical for the overall well-being and function of tissues to ensure the appropriate coordination and subsequent regulation of life processes.

Indeed, it was shown that when fibroblasts and epithelial cells were exposed to low streams of α-particles, their non-irradiated “bystander” counterparts growing in the same culture, were found to exhibit similar DNA damage responses (146). Furthermore, when cells were genetically compromised in their ability to participate in GJIC mediated by Cx43, they were no longer able to induce p21 expression after exposure to radiation, further demonstrating that the “bystander effect” occurs at least in part due to the inhibition of GJIC (146). Studies have demonstrated positive associations between GJIC and radiotherapy resistance in 3D culture conditions (147). Further supporting the hypothesis is the observation that restoration of Cx30 expression was able to reduce GBM cell growth while simultaneously conferring resistance to γ-radiation (148).

This resistance to therapy is particularly important when considered in the light of GJIC and cancer. Despite early evidence that connexins function as tumor suppressors, exceptions have been found in recent years showing that increased connexin expression may lead to tumors with more aggressive phenotypes (149). Evidence was first seen in melanoma cells transfected with cDNA coding for Cx26 which increased cellular metastatic capability in subcutaneous models of disease (116). The authors conjectured that this was due to a more effective way of facilitating cellular intravasation and extravasation, dependent on Cx26, which was found to aid GJIC between tumor cells, and normal endothelium (116). Multiple other studies have further confirmed that increased connexin expression within tumors can lead to a greater metastatic potential, migration, and invasion (150, 151).

It is not difficult to surmise that molecular mechanisms associated with GJIC and tumor suppressive roles are linked to particular signals that are exchanged among healthy cells and their cancerous counterparts. Furthermore, it should be understood that like healthy cells, tumor cells have the capability to interact with their microenvironment to affect their phenotype, although the exact contents of these interactions are still being puzzled out. Some molecules such as glutathione, a tripeptide with high permeability through GJ channels (152), have been shown to function as antioxidants to protect cells from ROS and DNA damage (153). While radiation is the most widely cited inducer of the “bystander effect” in cancer cells, additional molecules such as ROS, reactive nitrogen species (RNS), protein factors, and DNA molecules can also utilize GJIC to spread from the originally perturbed cell to damage its surrounding neighbors (154), although exosomal signaling, and hemichannel activity cannot be completely discounted as part of the mechanism. While it is commonly accepted that GJIC is one of the most important mechanisms behind the “bystander effect,” additional molecules such as necrosis factor-α (TNF-α) (155), transforming growth factor-β1 (TGF-β1) (156), interleukin-6 (IL-6) (157), IL-8 (158), and nitric oxide (NO) (159) have also been described, adding further complexity when considering cell-cell communication. In addition, individual connexins can have dual roles in the same tissue, acting as a tumor suppressors during primary progression while facilitating cancer growth in later stages of disease (17). Positive signals can also be released by targeted cells and spread to adjacent non-targeted cells to induce “bystander” responses. GJIC has therefore remained a critical component of normal physiology as well as of cancer biology and it is necessary to understand how the bystander effect may be manipulated to target tumor cells via GJs cancer therapeutics.

## GJIC and Epithelial-Mesenchymal Transition

There is broad consensus that epithelial-mesenchymal transition (EMT) involves epithelial cells losing their polarity and cell-cell contacts as they transition to a mesenchymal phenotype associated with highly invasive characteristics (160). The ability of tumor cells to disseminate and move from their original anatomical locations is closely associated with the occurrence of EMT and subsequent tumor malignancy. However, GJIC is also involved in cancer cell metastasis (161). Cx43 has been found to reverse epithelial-mesenchymal transition (EMT) and prevent resistance to cisplatin therapy in A549 lung adenocarcinoma cells (162), while increased adhesion and GJIC have long been known to play similarly critical roles in other highly metastatic lung carcinomas (163). The ability of tumor cells to communicate with their microenvironment is similarly implicated in EMT and metastasis. Increased GJIC between breast cancer cells and osteoblasts has been shown to make the former more metastatic compared to breast cancer cells that were only able to communicate between themselves. A decreased level of Cx43 was also associated with reduced GJs, promoting metastasis in MDA-MB-231 cells (161). In melanoma, in addition to GJIC, Cx26 expression was found to play a role in tumor cell intravasation and extravasation through communication with endothelial cells (116). Together, these observations suggest that cancer metastasis can be increased as a result of connexin expression and loss of GJIC (164). A potential explanation for this may be due to reduced connexin expression, which may allow for cells to physically detach from their substrates and undergo metastasis. However, in brain metastases from lung and breast tumors, human and mouse cancer cells expressed protocadherin 7, which promotes the assembly of GJs composed of Cx43 between tumor cells and astrocytes. Once cells were able to communicate, brain metastatic cancer cells were able to use GJ channels to transfer the second messenger cGAMP to astrocytes, activating inflammatory cytokine signaling pathways to promote tumor growth and chemoresistance (165). Thus, GJs can also facilitate brain metastasis formation, independent of EMT. GJs also play key roles as mediators of communication between cancer and endothelial cells, promoting tumor growth, and metastasis. An increasing body of work has confirmed that increased GJIC decreases metastatic dissemination in breast cancer and melanoma (164). In a study utilizing the metastatic breast cancer cell line MDA-MB-435 stably transfected with human Cx43 cDNA, Cx32 expression was reduced although GJIC, migration, and invasion were not affected (166). However, these cells showed decreased expression of N-cadherin, which is often associated with an aggressive cellular phenotype, as well as an increased sensitivity to apoptosis. Importantly, fewer lung metastases were shown in mice injected with MDA-MB-435 cells overexpressing Cx43, demonstrating that their metastatic potential could be blocked independently of GJIC (166). A potential mechanism for the contribution of GJIC to cancer cell adhesion and migration is thought to involve the physical interaction of GJs between tumor cells and other microenvironmental stroma, which prevents cancer cell dissemination (164). However, more work is necessary to identify how different connexin subunits function in metastasis, setting up the potential to develop GJ-enhancing agents to prevent tumor cells from spreading throughout the body.

## Hemichannels and Cancer Biology

Traditionally, GJs have been associated with cell-cell coupling and communication. As such, connexon hemichannels were first thought to be simple structural precursors to GJ channels before docking with their counterparts on adjacent cells (1). Unlike the investigation of GJIC, the quantification of connexin-mediated hemichannel activity presents with more challenges because multiple, unrelated mechanisms exist to facilitate how cells open membrane pores to communicate with their extracellular environment. Evidence for functional connexon hemichannel activity was first elucidated in catfish retina cells due to their permeability to Lucifer dye (167). Further studies sought to better define the particular connexin subunits responsible for the formation of permeable channels in cells. Upon induction of Cx46 expression in *Xenopus* oocytes, it was found that cells similarly became water permeable and underwent lysis unless osmotically buffered with Ficoll (28). While initial observations reached the conclusion that hemichannels remained closed until connexons docked with one another to form GJs, subsequent work would describe a variety of different regulatory mechanisms to facilitate pore activity. Among these are intracellular and extracellular factors such as changes in the ionic concentration of the microenvironment, in particular Ca^2+^ gradients (32), although Na^+^, and K^+^ have also been implicated in hemichannel regulation (168). Membrane depolarization has also been found to induce single hemichannel opening in HeLa cells engineered to express Cx43 (169). Likewise, metabolic inhibition was sufficient to open heterologously expressed Cx43 hemichannels in cardiac cells upon exposure to calcium-free media conditions (170). However, differences between ionic concentrations in cells and their microenvironment are not the only method by which hemichannel activity can be affected, as fluid flow shear stress was found to induce Cx43 translocation to osteocyte membrane surfaces to serve as a release mechanism for prostaglandin E2 in response to mechanical strain (171).

With the use of connexin mutants that result in dead channels, as well as pharmacological inhibitors of GJIC, it has been determined that connexins display their tumor suppressive properties in a hemichannel-dependent manner (172, 173). The mechanisms by which this occurs are still in the early stages of investigation, although it has been noted that the tumor suppressive capabilities of Cx43 in keratinocytes may be linked to its interaction with caveolin 1, another factor associated with tumor suppression (174). Moreover, Cx43 expression has also been implicated in prostate cancer and is correlated with its metastatic potential although only direct Cx43 knockdown but not GJ channel formation was seen to decrease cell migration and invasion, indicating that hemichannel activity was critical for cellular function (117). The cytoplasmic C-terminal domain of Cx43 was also shown to be sufficient in suppressing neuroblastoma progression via Src signaling (175). Interestingly, connexin hemichannel activity was found to be crucial for the regulation of the actin cytoskeleton in human glioma cells (176). Using time-lapse microscopy, Cx43 levels were shown to delay mitotic duration, corresponding with an accumulation of cells in G1, further leading to increased levels of p21waf1/cip1, a cell cycle inhibitor (177), suggesting that the upregulation of Cx43 delays the cell cycle rate through the delay of G1, pointing to more roles relating to the GJIC-independent function of connexins.

Numerous studies now indicate that connexin-mediated hemichannel activity plays an integral role in cell-microenvironment communication in a variety of different tissues and aspects of cell life (178). Functional studies have demonstrated that hemichannels play important roles in Ca^2+^ signaling (179), cell proliferation (180), and apoptosis (181), as well as the normal development of a variety of cell types (182). For instance, neurite outgrowth in PC12 cells, which are derived from a pheochromocytoma of the rat adrenal medulla and used to study neuronal development (183), was found to be mediated by hemichannels after stimulation with nerve growth factors. Furthermore, it was found that hemichannel-mediated ATP release and its subsequent interaction with purinergic receptors was sufficient to induce growth and neuronal differentiation in the same cell line (184). Likewise, Cx43 hemichannels have been implicated in the back and forth movement of NAD+, which is thought to regulate Ca^2+^ gradients via CD38 transmembrane glycoproteins in 3T3 fibroblasts (185). In heart ventricular myocytes, hemichannel activity has been described and shown to have osmoregulatory properties, which have both positive and negative impacts on myocardial infarctions and normal cardiac physiology (186).

In general, connexin hemichannels in the open position are thought to be detrimental to cells due to their impact on the efficiency of cell metabolism and the maintenance of the ionic balance between the interior and exterior of cellular membranes (178). If hemichannels remain open to allow the passive diffusion of extracellular material into the cell or the egress of cytoplasmic contents into the extracellular environment, cells will not be able to sustain normal homeostasis, and will thus undergo cell death. Open hemichannels can be thought to behave as pathogenic pores, as they play important balancing roles between cell death as a result of necrosis or apoptosis via the controlled release of ATP (187). The depletion of cellular ATP may also activate connexin hemichannels, creating a feedback loop by opening otherwise closed channels (188). In an oft-cited example, staurosporine, an ATP-competitive kinase inhibitor, was shown to open Cx43 hemichannels and induce apoptosis, which was itself inhibited by truncating the C-terminal tail of the connexin, thereby forming non-functional hemichannels (189). However, other studies have demonstrated that closure of connexin hemichannels may reduce the development of apoptosis. When HeLa cells, which are deficient in coupling ability, were transduced with wild-type Cx32 and Cx43, apoptosis was increased following treatment with variety of cytotoxic agents.

Following hemichannel closure via pharmacological means, apoptosis was found to be slowed, demonstrating the role of hemichannels in microenvironmental communication and cell function (190). In contrast, alendronate, a bisphosphonate drug used to treat osteoporosis, was found to be capable of inhibiting apoptosis by opening rather than closing Cx43 hemichannels, which in turn activated Src kinase and Erk, promoting cell survival (191). In other organ systems such as the heart, release of ATP through different methods, including connexin hemichannels, has been demonstrated to be a stress response that is capable of vasodilation and the facilitation of increased delivery of oxygen, and energy (192). Thus, continued release of ATP would result in harmful consequences and eventually lead to cell death. As such, phosphorylation of mitochondrial Cx43 has been implicated in Cx43 hemichannel communication and cardioprotection (193). In tissues of the nervous system, connexin hemichannels can play both protective and harmful roles via diffusion of necrotic or apoptotic signals from injured cells to healthy ones or by allowing for the diffusion of ions and protective signals from healthy to injured cells (194, 195). Thus, it becomes imperative to understand whether pharmaceutical tools can be used for cardio-and neuro-protection by targeting specific connexin hemichannels. While nonspecific GJ inhibitors such as halothane, 1-octanol, carbenoxolone, and mefloquine may reduce injury in certain animal models, the need to characterize connexin hemichannel function and its roles in ATP release or Ca^2+^ signaling remains under-studied in cancer. Thus, designing new and specific connexin mimetic peptides may serve as a promising and strategic means by which adjacent cells can be protected from injury as a result of myocardial infarction, stroke, or cancer for which cellular communication has an important component.

## Connexins, Their Protein Partners, and Cancer

Connexins have traditionally been associated with communication, whether through GJIC or through hemichannel formation. Increasing evidence however, supports GJ-independent roles for connexins through a diverse set of interacting protein partners (196). Among the first of such reports, Cx26 was shown to suppress tumor-derived mammary epithelial cells (197). The induced expression of Cx26 in GJ-deficient MCF-7 breast cancer cells also resulted in decreased proliferation, invasion, and *in-vivo* tumor growth, although their communicative capacity has not yet been investigated (198). However, this did raise interesting questions regarding the role of connexin proteins in tumor growth. Subsequent studies confirmed and further demonstrated that Cx26 can inhibit breast cancer cell migration and overall tumorigenesis in the MDA-MD-435 tumor cell line independent of GJ function. The likely mechanism was determined to result from the regulation of β1-integrin and MMP levels, indicating that communication was not the sole function of the connexins (97). Further work in triple-negative breast cancer (TNBC) revealed that Cx26 is capable of interacting with NANOG and focal adhesion kinase (FAK) to drive tumor progression and CSC self-renewal (99). Other connexin subunits have also been assayed for their tumor suppressive function via interaction partners. Cx32 was found to decrease tumor growth, invasion, and metastasis of renal cell carcinoma cell lines via multiple modulators, including Src, tight junction proteins, and vascular endothelial growth factor (VEGF), independent of GJIC function (199). In addition, it has long since been established that ectopic expression of a Cx43 mutant without intrinsic GJ function is able to prevent cell growth through the association of its cytoplasmic carboxyl domain with proteins such as ZO-1 and c-Src (175). It has also been demonstrated that alteration of connexin levels, either through forced expression or deletion, can lead to changes in downstream gene expression in seemingly unrelated pathways. Cx43 deletions were studied in the context of astrocytes in the neonatal brain, and it was found that large numbers of genes were statistically changed in mice with decreased expression of the connexin (200). Moreover, when two Cx43 mutants were created, one without the C-terminal domain and one without the entire transmembrane domain, a reduction in glioma proliferation was described (176). Additionally, truncation of Cx43 did not alter GJ coupling, and it was demonstrated that the Cx43 C-terminal domain was sufficient to induce glioma cell migration, which was associated with a lamellipodia-type migration and actin cytoskeleton regulation (176). It has also been shown that Cx43 is associated with increased sensitivity to sunitinib-induced cytotoxicity in malignant mesothelioma cells, an effect that is independent of channel formation but is rather a result of its interaction with the apoptotic factor Bax (201).

The mechanisms behind GJ-independent connexin function are still being investigated. One proposed answer involves connexin-responsive elements (CxRE), which are hypothesized to induce differential recruitment of sp1 and sp3 transcription factors to the CxRE via the ERK/PI3K pathway (202). Thus, the functional consequence of such mechanisms is the regulation of genes that have the promoter element and respond to differential connexin regulation. Indeed, the effect of connexins on gene expression has been investigated in multiple studies wherein re-expression of connexins in deficient tumors is sufficient to affect their characteristics, namely tumorigenicity, which cannot simply be the result of cytoplasmic exchange (1). Cell differentiation has also been implicated in GJ-independent functions of connexins as evidenced by the ability of Cx45.6, but not Cx43 or Cx56, to stimulate lens cell formation regardless of its ability to form GJ channels. Furthermore, it was demonstrated that the C-terminal domain of Cx45.6 was, by itself, enough to induce lens cell differentiation (203). Additionally, Cx43 was shown to control the directional motility of cardiac neural crest cells via the actin-binding proteins vinculin and drebrin (204), demonstrating that connexins should not only be thought of in the context of cellular communication but should rather be considered in a cell-type specific manner. Adding weight to such conclusions are observations that embryonic neurons are able to migrate using Cx43 and Cx26, as shown via knockout mouse models, which provide cytoskeletal contacts with radial fibers without the exchange of cytoplasmic contents (205). Thus, our understanding of connexin function is evolving as individual cells and tissues are interrogated. No longer are connexins only studied in the context of cell-cell communication, but rather they are known to be involved in a wider milieu of disparate and seemingly unrelated processes. Additional work is necessary to define how connexins are able to carry out each of their roles and whether one function is more important than the others in certain contexts. However, a GJ-independent role for connexins opens up a world of novel observations that could be critical for normal physiology, pathology, and therapeutics.

## Concluding Remarks

Over the past 50 years, remarkable work has been conducted to investigate how connexins function in cell-cell communication, hemichannel activity, and other activities unrelated to GJIC. Proper cellular adhesion and communication are necessary for the development of multicellular life, without which it would not be possible to coordinate larger-scale behavior and cellular responses. Unlike other junctional molecules, connexins help enable tissue organization and mediate the transfer of signals among cells during development, maintenance of homeostasis, and pathology. Thus, understanding their biology and regulatory mechanisms at the transcriptional and translational levels will enable additional exploration regarding their large repertoire of functions. In fact, connexins may have additional thus-far undefined roles in cellular development, differentiation, and physiology outside of the scope of this review. It is of utmost importance to study aberrant connexin function as it relates to disease states including cancer. Likewise, it is critical to remember that connexins do not exist in a vacuum. Rather, their characterization should be considered in temporal and tissue-specific contexts. While one connexin subunit may be required for the growth of a particular tissue, it should not be assumed that this will hold true throughout the lifetime of the organism.

The recent push to identify GJ-independent functions of connexins, whether they take the form of hemichannels or connexin-protein interactions, has given rise to novel questions about their role in normal cell physiology. However, their complexity and overall tissue distribution has made it difficult to fully elucidate their function in human development. It should also be mentioned that while there is a wide range of knowledge about the specific types of molecules that are shuttled via connexins, as of yet, it is difficult to definitively state how individual connexin subunits are selectively permeable to particular signals. That is not to say that such efforts are fruitless but that this should be a larger area of investigation to potentially identify novel means to deliver drugs, chemotherapy, or other pharmacological agents into cells via GJs. While there is still a great deal to discover regarding GJIC, advances are rapidly gaining momentum to answer such questions.

Connexins and GJIC are increasingly attractive targets for cancer therapy as their functions become better defined. Arguably, the most advantageous feature of anti-connexin strategies is the ubiquitous nature of the proteins. Most normal cells of the body require the ability to communicate in order to carry out tissue and organ-level functions that require precise regulation and rapid response to changing local and systemic conditions. Thus, connexin trafficking and turnover are tightly controlled, as suggested by their rapid half-lives. Such properties of connexins and GJIC enable cells to quickly generate correct connections with their neighbors or microenvironment and coordinate large-scale actions that would not be possible on a single-cell level. Moreover, tumor cells are also able to co-opt such function to facilitate their sustained growth. Intercellular communication is especially important in the context of cancer because tumors should not be thought of as simply an amalgamation of rapidly proliferating cells but rather as discrete entities that are able to manipulate their microenvironment to create conditions that are more conducive for survival.

While the connexin family is composed of over 20 distinct subunits, tumors likely express only a few isoforms for the facilitation of GJIC. Thus, with proper investigation, including bioinformatics and functional studies, connexins could serve as tumor markers based on the pattern of expression on cancer cells. Additionally, their conserved transmembrane regions make connexins and GJs sensitive to a wide variety of different pharmacological inhibitors. Currently several agents with pan GJ inhibition activity, including some that are FDA-approved for unrelated conditions, are widely utilized, including carbenoxolone, 1-octanol, mefloquine, halothane, histamine, and others [as reviewed in Salameh and Dhein (206)]. However, while these agents are known to be capable of disrupting GJIC and hemichannel function, it is more difficult to understand how connexin-protein interactions are affected due to the intracellular C-terminal domain. Whereas, blocking certain GJs on tumor cells could have positive consequences in terms of hindering proliferation and growth, deleterious side-effects may also occur as a result of inhibition of GJIC on normal cells making it is necessary to explore peptides that are able to inhibit specific connexins to selectively target tumor-specific proteins while sparing those utilized by healthy, non-cancerous cells. While some connexin-specific peptides such as Gap19 for Cx43, Gap27/40 for Cx40, and Gap 24 for Cx32 [reviewed by Evans and Leybaert (207)] exist, their specificity and mechanism of action are still under scrutiny. Likewise, their efficacy in animal settings is still being elucidated, and it is necessary to better understand their pharmacology, toxicity, and anti-tumor function. In a recent review by Laird and Lampe, the authors provide a detailed summary of current ongoing clinical trials utilizing connexin-based therapy for the treatment of epidermal injury, eye wounds, and inflammation while detailing the challenges posed by connexin therapeutics in human clinical trials (208).

It is also important to consider which of the three aforementioned functions of a connexin is most appropriate for targeting strategies. Table 2 summarizes which connexins are pro-or anti-tumorigenic in the context of the three main functions described in this review. Blocking or otherwise inhibiting GJIC and hemichannel function is the most straightforward method, as it forces cells to remain in relative isolation and unable to respond to harmful internal or external conditions. It has been found that a Cx43-specific peptide, L2, is capable of keeping Cx43 GJs in an open state while inhibiting hemichannel opening to investigate the therapeutic potential of counteracting excessive activity without disrupting GJIC (209). Thus, such tools will enable the development of strategies toward specific connexin functions in a more personalized manner. This is most evident when CSC or tumor cell communication is inhibited prior to chemotherapy administration, increasing their sensitivity to current standard-of-care practices, likely as a result of ROS accumulation or the inability to shuttle out toxic molecules (96, 113). However, inhibiting connexin-protein interactions, while arguably more difficult, could lead to more specific anti-tumor approaches, as only certain proteins are able to associate with connexins within particular neoplasms (99). Blocking such contacts could affect critical downstream signaling pathways including PKC, MAPK, and Src to induce tumor cell death while sparing normal cells lacking the connexin-protein complex. That is not to say that only those three connexin functions are attractive targeting opportunities, but the end result would still disrupt proper connexin function. Blocking connexin biosynthesis or trafficking could also represent valid anti-tumor strategies, although this would still lead to GJ, hemichannel, or connexin-protein interaction dysfunction. Thus, in order to consider how connexins may be utilized as potential therapeutic targets in cancer, one must take into account their functional diversity and cellular specificity. Whereas, targeting GJIC in one tumor type could limit proliferation or induce death, the same cannot be assumed to be true across different malignancies. The same concept applies to hemichannel activity and connexin-protein interactions, although they do allow for more specificity when targeted. Moreover, it should also be remembered that targeting a single connexin subunit may simply not be sufficient to cause tumor cell death, as different subunits are able to compensate for the loss of a single group of channels. Thus, careful consideration is warranted when designing connexin and GJIC-mediated therapy. However, this is not a reason to abandon targeting intercellular communication in cancer but rather a promising area of development. By inhibiting tumor cell communication, fewer and smaller doses of chemotherapy can be applied, limiting harmful side effects while still retaining efficacy against tumor cells. Ideally, inhibiting connexin function will synergize with current standard-of-care therapies to enable treatment where other options are not available. Thus, connexin function is a crucial, multifaceted process that may enable next-generation anti-tumor modalities.

## Author Contributions

All authors listed have made a substantial, direct and intellectual contribution to the work, and approved it for publication.

### Conflict of Interest Statement

The authors declare that the research was conducted in the absence of any commercial or financial relationships that could be construed as a potential conflict of interest.
